# Urinary Tract Infection Predictors in Patients Undergoing Retrograde IntraRenal Surgery for Renal Stones: Does the Instrument Make the Difference?

**DOI:** 10.3390/jcm13102758

**Published:** 2024-05-08

**Authors:** Francesco Prata, Loris Cacciatore, Annamaria Salerno, Francesco Tedesco, Alberto Ragusa, Salvatore Basile, Andrea Iannuzzi, Antonio Testa, Gianluigi Raso, Giuseppe D’Addurno, Marco Fantozzi, Marco Ricci, Antonio Minore, Angelo Civitella, Roberto Mario Scarpa, Rocco Papalia

**Affiliations:** Department of Urology, Fondazione Policlinico Universitario Campus Bio-Medico di Roma, 00128 Rome, Italy; francesco.prata@gmail.com (F.P.); annamaria.salerno@policlinicocampus.it (A.S.); francesco.tedesco@unicampus.it (F.T.); alberto.ragusa@unicampus.it (A.R.); salvatore.basile@unicampus.it (S.B.); andrea.iannuzzi@unicampus.it (A.I.); antonio.testa@unicampus.it (A.T.); gianluigi.raso@unicampus.it (G.R.); giuseppe.daddurno@unicampus.it (G.D.); marco.fantozzi@unicampus.it (M.F.); ma.ricci@unicampus.it (M.R.); antonio.minore@unicampus.it (A.M.); a.civitella@policlinicocampus.it (A.C.); r.scarpa@policlinicocampus.it (R.M.S.); rocco.papalia@policlinicocampus.it (R.P.)

**Keywords:** UTI, urinary infections, single-use, multi-use, ureterorenoscopy, RIRS, predictors

## Abstract

**Background:** Retrograde intrarenal surgery (RIRS) using flexible ureterorenoscopes is a cornerstone approach for renal stone removal, yet it carries a significant risk of postoperative urinary tract infection (UTI). With the emergence of single-use ureterorenoscopes, there is growing interest in their potential to mitigate this risk. This study aimed to compare the postoperative infection rates between single-use and multi-use ureterorenoscopes in RIRS procedures and to identify predictors of postoperative UTI. **Methods:** Data were collected from 112 consecutive patients who underwent RIRS for renal stones between March 2022 and September 2023. Peri-operative variables including age, gender, body mass index (BMI), stone size, stone location, type of ureterorenoscope, Hounsfield Units (HU), pre-operative hydronephrosis, laboratory analysis, and operative time were evaluated. Univariate and multivariate logistic regression analyses were performed to assess the predictors of postoperative UTI. **Results:** Of the cohort, 77 surgeries (68.7%) utilized multi-use ureterorenoscopes, while 35 (31.3%) utilized single-use devices. Stone diameter, number of stones, type of ureterorenoscope, and operative time were significant predictors of postoperative UTI in the univariate analysis. Multivariable logistic regression showed that operative time (OR, 1.3; 95% CI, 0.55–0.99; *p* = 0.03) and type of ureterorenoscope (multi-use vs. single-use) (OR, 1.14; 95% CI, 1.08–1.2; *p* < 0.001) were independent predictors of postoperative UTI. **Conclusions:** In conclusion, this study highlights that multi-use ureterorenoscopes and prolonged operative time are associated with an increased risk of postoperative UTI in RIRS procedures. Careful pre-operative evaluation and meticulous patient selection are essential to minimize the occurrence of postoperative UTIs and optimize patient outcomes in RIRS for renal stones.

## 1. Introduction

The prevalence and incidence of nephrolithiasis are reported to be increasing across the world [1]. This rise coincides with significant advancements in surgical technology, including endoscope miniaturization, improved deflection mechanisms, enhanced optical quality, and the development of advanced tools. These technical improvements have propelled retrograde intrarenal surgery (RIRS) to the forefront of stone management, contributing to its substantial progress and widespread adoption [2]. This treatment, facilitated by a flexible ureterorenoscope, stands as a pivotal technique for renal stone removal, offering remarkable efficacy and minimal invasiveness [3,4].

The endorsement of RIRS as a preferred approach for managing certain types of renal stones by the European Association of Urology (EAU) guidelines underscores its importance, particularly considering factors such as stone dimensions and position [2,5]. However, the application of RIRS often faces the challenge of a notable incidence of postoperative urinary tract infections (UTIs), which carry the potential for harmful complications [6]. More in detail, a systematic review reported that postoperative UTIs were observed in 3.9% of rigid or flexible ureteroscopies [7]. Among these UTIs, 6.5% were classified as urosepsis, underlying how these procedures can lead to severe and detrimental consequences when risks are underestimated.

Recent studies reported that some outbreaks have been linked to contaminated instruments resulting from inadequacies in the sterilization process [8,9]. In response to this concern, single-use ureterorenoscopes have emerged as a solution, garnering increasing attention and adoption within stone management centers worldwide. These disposable instruments not only promise to mitigate the risk of cross-contamination but also hold potential in addressing the persisting issue of postoperative infections [10].

In the current literature, several systematic reviews have focused their attention on the overall procedural costs between disposable and reusable scopes, hypothesizing that in high-volume centers, reusable ones are likely to be cost-effective [11,12]. On the other hand, concerning efficacy and postoperative complications, there are many small-, medium-, and high-volume prospective studies that have reported on the infectious complications following URS for renal stone disease [13,14,15,16]. Some studies have also looked at the risk factors for urinary infections following ureteroscopy and advised on strategies to reduce these risks [7]. However, the granularity of the data available prevents us from establishing the real net benefit of single-use ureteroscopes. On this background, this study seeks to evaluate the postoperative infection rates in patients undergoing RIRS with single-use flexible ureterorenoscopes compared to their multi-use counterparts, offering a comprehensive analysis of the contemporary challenges and opportunities in renal stone surgery. Furthermore, the investigation aims to identify predictors of postoperative UTIs, shedding light on factors crucial for optimizing patient outcomes and procedural efficacy.

## 2. Materials and Methods

### 2.1. Patient Population

In accordance with the Declaration of Helsinki, between March 2022 and September 2023, we conducted a retrospective analysis in patients diagnosed with renal stones who underwent RIRS with single- and multi-use ureterorenoscopes at our institution. All surgeries were conducted by two experienced (over ten years and over 500 procedures performed) surgeons and respected the same procedure with the help of X-rays; Table 1 shows the used devices and their characteristics. Patients with ureteral stents and/or those who underwent a second-look RIRS or had missing data were excluded from the study. Patients’ demographic, anthropometric, and clinical characteristics at baseline, such as age, gender, and body mass index (BMI), were collected. All patients underwent a standardized preoperative work-up, which included an electrocardiogram and cardiology consultation; chest X-ray; comprehensive blood tests comprising complete blood count with white cell differential, creatinine, blood urea nitrogen, and electrolyte assessments; urine analysis and urine culture; as well as anesthesiology evaluation. All patients underwent preoperative contrast-enhanced computed tomography (CT) scans specifically tailored for urological evaluation (URO-CT) within three months from surgery. These scans provided a detailed assessment of stone-related characteristics, including size, location, Hounsfield Units (HU), and grade of hydronephrosis, for an accurate preoperative planning and intraoperative guidance during ureterorenoscopy. Comprehensive peri-operative data were meticulously collected to facilitate a thorough evaluation, including the type of ureterorenoscope, type of energy, operative time, and placement of ureteral stenting. After treatment, pre- and postoperative laboratory tests (blood and urine) were compared.

### 2.2. Urinary Tract Infection Assessment

To prevent infection following ureteroscopy stone removal, according to EAU guidelines [2,17], a preoperative urinalysis was conducted for all patients. In instances of positive results, a reflex urine culture was promptly initiated and treated in accordance with the urine antibiogram. Perioperative antibiotic administration adhered to the best-practice guidelines established by the EAU, incorporating the use of empiric first-generation cephalosporin or personalized antibiotics guided by past allergic reactions or culture/sensitivity data whenever available. Routine postoperative beta-lactam antibiotic prophylaxis with or without beta-lactamase inhibitor was administered for a total duration of 5 days, with first taking it in the hospital following the procedure and subsequently at home after discharge.

Between 15–20 days following the endoscopic intervention, all patients underwent urine culture. If the culture yielded positive results, patients received a personalized antibiotic treatment tailored to the specific bacteria identified.

### 2.3. Follow-Up

Following ureteroscopic treatment, a meticulous follow-up regimen is essential to supervise patient recovery and measure the effectiveness of the intervention. This involves a structured approach:

1. Within 3–4 weeks after the procedure, patients are evaluated at a dedicated urological outpatient clinic catering specifically to stone-forming patients. Symptoms reported by the patient, such as persistent pain, urinary urgency, or hematuria, are meticulously documented during follow-up visits, as they may indicate underlying complications or a recurrence of stones.
2. Imaging modalities such as renal ultrasound, X-rays, or CT scans are employed to assess the stone-free rate and verify the correct placement of ureteral stenting. Routine urine analysis and culture are also conducted to monitor urinary tract infections and assess the effectiveness of any prescribed antibiotic regimens.
3. If the imaging results are satisfactory, patients may undergo ureteral stent removal in an outpatient cystoscopic setting. This step aims to facilitate healing and improve patient comfort.
4. Any stone fragments retrieved during the procedure undergo compositional analysis, including a spectrophotometric analysis and metabolic evaluation conducted in collaboration with an endocrinologist. This comprehensive assessment aids in determining the stone composition and guides the formulation of preventive strategies to mitigate the risk of future stone formation.
5. Patients undergo extensive education on dietary modifications, hydration strategies, and medication adherence during dedicated nutritional counseling sessions. These measures are designed to proactively address potential risk factors and promote urinary tract health.
6. Long-term surveillance appointments are scheduled based on the individual patient’s risk profile and stone history. These appointments typically include renal ultrasound examinations at 6–12-month intervals, supplemented by periodic blood tests.

### 2.4. Statistical Analysis

Continuous variables are presented as medians and interquartile ranges (IQRs), while categoric variables are presented with frequencies and proportions. To investigate the predictors of postoperative urinary tract infection (UTI) following RIRS, both univariate and multivariate logistic regression analyses were performed. A two-sided *p*-value < 0.05 was established as the threshold for statistical significance, ensuring robust assessment and interpretation of the findings. Statistical analyses were conducted using STATA (StataCorp. 2021. Stata Statistical Software: Release 17. StataCorp LLC.: College Station, TX, USA).

## 3. Results

**Figure 1:**
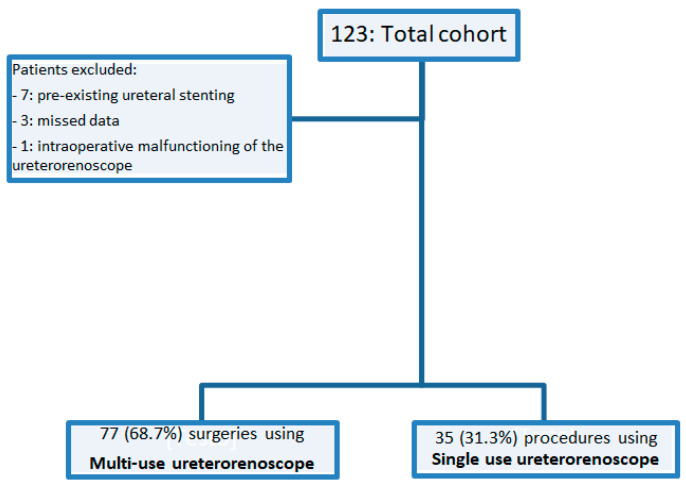
Flow chart.

After the first retrospective evaluation, we excluded seven patients with pre-existing ureteral stenting and three patients with missing data. Additionally, after treatments, one patient was excluded from the analysis due to intraoperative malfunctioning of the ureterorenoscope (*n* = 1). Out of 123 cases, we obtained a final cohort of 112 cases, as shown in Figure 1 (flow diagram). Of the total count considered, 77 surgeries (68.7%) were performed using a multi-use ureterorenoscope, while 35 procedures (31.3%) utilized a single-use ureterorenoscope. Table 2 shows our cohort characteristics. The median age and BMI of the cohort were 58 years (IQR, 50–66) and 26.12 kg/m^2^ (IQR, 23.74–29.39), respectively. Left renal stones were observed in the majority of patients (58%), followed by right renal stones in 39% of cases and bilateral lithiasis in only 3% of cases. The median stone size and Hounsfield Units (HU) were 12 mm (IQR, 9.5–17.5) and 1350 (IQR, 950–1640), respectively. The majority of procedures utilized Holmium laser lithotripsy (69.9%), while 30.1% underwent lithotripsy with Thulio^®^ utilizing RealPulse technology (Dornier, MedTech). The median operative time was 57 min (IQR 35–85). Following the endoscopic procedures, 25 patients (22.3%) developed postoperative Gram-negative UTIs.

Table 3 reports the logistic regression analysis. More in detail, univariate logistic regression analysis revealed that stone diameter (OR, 1.15; 95% CI, 1.05–1.25; *p* = 0.001), number of stones (OR, 2.24; 95% CI, 1.25–4.01; *p* = 0.006), type of ureterorenoscope (multi-use vs. single-use) (OR, 1.28; 95% CI, 0.55–0.97; *p* = 0.01), and operative time (OR, 1.13; 95% CI, 1.08–1.18; *p* < 0.001) were predictors of postoperative UTI. In the multivariable logistic regression analysis, only operative time (OR, 1.3; 95% CI, 0.55–0.99; *p* = 0.03) and type of ureterorenoscope (multi-use vs. single-use) (OR, 1.14; 95% CI, 1.08–1.2; *p* < 0.001) were independent predictors of postoperative UTI.

## 4. Discussion

Since 1912, when Hugh Young mistakenly introduced a rigid cystoscope into a pediatric patient’s ureter, leading to the birth of ureteroscopy, this instrumentation has facilitated the diagnosis and treatment of various urological diseases [18]. Subsequently, in 1964, Marshall’s fiber optic ureteroscope project marked the beginning of several instrumental improvements, in particular with regard to image quality, durability, and performance, up to the present day with the advent of disposable ureteroscopes [19]. The comparison between single-use and multi-use ureteroscopes encompasses two critical factors: costs and environmental impact [20]. Regarding costs, they vary significantly depending on the geographic location (e.g., in France, the flex X2 ureteroscope costs GBP 17,000; in Germany, it costs GBP 13,200; whereas in Turkey, it costs GBP 29,500) [21]. However, costs are not solely determined by the price of the individual instrument but also encompass decontamination, transportation, storage, and repair expenses, which fluctuate based on the number of procedures performed [22,23]. On the other hand, in terms of environmental impact, the comparison involves evaluating the production and disposal of CO_2_ emissions and waste. Specifically, a study on the comparison between single-use and multi-use flexible cystoscopes highlighted that the total water consumption during procedures and the reprocessing of reusable devices amounted to 800 g per procedure, whereas for single-use devices, it was only 200 g [22].

Although the indications for single-use ureteroscopes may be vast and could serve as a reasonable alternative for patients to reduce the infection risk, the multi-use one is still the most widely utilized in the hospitals [24]. Among the various postoperative complications, UTIs associated with urinary stone disease and endoscopic procedures, such as ureterorenoscopy (URS), stand out as notable challenges in contemporary urological practice [7]. Indeed, UTIs represent one of the most prevalent bacterial infections affecting individuals across the globe, with significant implications for patient morbidity, antimicrobial resistance, and healthcare costs [25,26]. The interaction between urinary stones and bacterial colonization provides a fertile ground for the development of UTIs, as the presence of calculi can lead to urinary stasis, obstruction, and mucosal trauma, predisposing patients to microbial ascent and infection [27,28]. Recent studies in line with those reported from the EAU guidelines [17] offer valuable insights into the management of UTIs in urological practice, emphasizing the importance of risk stratification, perioperative antibiotic prophylaxis, and tailored antimicrobial therapy based on local resistance patterns and patient-specific factors [29,30].

Nowadays, the availability of novel technologies, such as single-use ureteroscopy and advanced ureteral access sheaths, has opened doors to less-invasive procedures. These innovations hold the promise of minimizing patient morbidity while potentially reducing the incidence of UTIs. Against this backdrop, we investigated the incidence of postoperative infections among patients undergoing RIRS with single-use flexible ureterorenoscopes in comparison to those utilizing multi-use counterparts. Our study provides a detailed analysis of the current landscape, shedding light on the challenges and prospects in renal stone surgery showing interesting findings.

We reported a 22.3% rate of postoperative UTIs occurring in patients who underwent endoscopic procedure, which is consistent with rates in similar studies reported by Mitsuzuka et al. (18.3%) [31] and by El-Agamy et al. (15.6%) [29]. In the univariable logistic regression analysis, several factors associated with postoperative UTI, including stone diameter, number of stones, type of ureterorenoscope (multi-use vs. single-use), and operative time, were evaluated. These findings are in line with those in the current literature, suggesting that larger stone size, increased stone burden, prolonged operative time, and certain instrumentation factors may contribute to a higher risk of postoperative UTIs. More in detail, these results seems comparable with those reported by Shreya Chugh et al. in their systematic review [7]. While the univariate analysis identified stone number and volume as predisposing factors, they did not emerge as particularly significant in the subsequent multivariable logistic regression analysis, where only operative time and the type of ureterorenoscope emerged as the independent predictors of postoperative UTIs. These findings underscore the significance of these variables in influencing outcomes as well as the importance of optimizing surgical efficiency and selecting appropriate instrumentation in order to reduce the development of urinary infections.

Concerning the difference between single- and multi-use ureteroscopes, the latter is the most widely used but requires reprocessing and sterilization after each procedure. These processes can subject the instrument to the risk of breakage, blurred optics, and worsening endoscopic vision. Moreover, several factors, including the type and duration of the procedure, surgeon experience, and prior ureteroscope refurbishment, contribute to the reduced durability of reusable ureteroscopes [32]. On the contrary, disposable instruments have the great advantage of not requiring maintenance processes, ensuring a high performance of vision and effectiveness, thus customizing the instrument for the patient. In addition, another great advantage is to wipe out the risk of bacterial contamination and transmission from one patient to another [13,33,34]. Therefore, inadequately sterilized instruments can serve as a source of infection and may be transmitted to multiple patients.

The transfer of bacteria from one host to another presents an important concern to reusable instrumentation. Despite major improvements in sterilization processes [35], communicable outbreaks are still described globally due to improper disinfection procedures, such as sub-optimal reprocessing by staff or the development of bacterial resistance against specific detergents, as observed in some studies. In the study by J. Kumarage et al., the improper sterilization of two flexible ureteroscopes in a healthcare center in the United Kingdom resulted in the infection of 13 patients with a strain of antibiotic-resistant Pseudomonas aeruginosa [8], while Adam Cole et al. reported that the rate of hospital readmission due to instrument-related infection was 2% of patients [9]. These findings underscore the critical nature of UTIs as a significant complication of healthcare procedures. Among the multi-resistant infections, outbreaks of Enterobacter cloacae and Pseudomonas aeruginosa were mainly detected [36]. A more recent ex vivo chemical analysis of ureteroscopes in two major U.S. centers reported 13% microbial growth on sterilized instruments and 100% protein on devices. Regardless of manual cleaning and sterilization with hydrogen peroxide, problems were related to structural irregularities, discoloration, or damage to the ureteroscope operating channel [37]. All of these conditions lead to high maintenance costs, especially in high-volume ureterorenoscopy centers, as reported by several studies. Indeed, each reprocessing event was estimated to cost about USD 96.13 [38], without considering the repair cost. Concerning repair processes, they were calculated to offer between 4.75 and 7.7 uses for older models [39], with an approximately USD 9420 per each repair cost [38,40]. Therefore, to the initial purchase price, the maintenance costs associated with depreciating ureteroscopes must also be considered. Nonetheless, these costs may be avoided by the use of single-use ureteroscopes.

On the other hand, among various predisposing factors for urinary tract infections (UTIs), operative time was identified as a predictor in a research study. This finding aligns with previous research by Chugh et al. and Baboudjian et al., who also suggested that minimizing operative times could potentially decrease postoperative infection rates [7,30].

Notwithstanding these results, our investigation is not devoid of limitations. Firstly, a retrospective analysis from a single center may limit the generalizability of the findings to other settings. Variability in patient populations, surgical techniques, and postoperative care protocols across different institutions could influence the results. Moreover, our dataset did not provide information regarding the impact of two different instruments (single- vs. multi-use) on the stone-free rate and hospital costs. Additionally, the metabolic values, such as parathyroid hormone or Vitamin D, were not provided. Furthermore, the duration of follow-up is a restricted period to evaluate the different postoperative management chosen. Finally, our study lacks the comorbidities serving as potential confounding factors to adjust with in our multivariate analysis.

Despite these limitations, our study lays the groundwork for future research and still offers valuable insights into the field of UTIs, suggesting potential perioperative management strategies such as using single-use ureteroscopes and minimizing operative time.

## 5. Conclusions

Our study suggests that in retrograde endoscopic treatment for renal stones, the use of multi-use ureterorenoscopes and longer operative times are associated with an increased risk of postoperative UTIs. Implementing a single-use instrumentation approach could potentially mitigate this infectious risk, particularly in patients with higher infectious risk and more complex stones. While the initial cost of disposable instruments may pose a challenge, their benefits may outweigh the costs in select patient populations. High-volume centers like ours may benefit from a hybrid approach, incorporating both single- and multi-use instruments to balance economic and environmental considerations. Further prospective, multicentric studies are warranted to validate these findings and refine strategies for reducing UTI-related complications in ureteroscopy.

## Figures and Tables

**Table 1:** Used devices during RIRS procedure.

Devices	Length/Size/Thickness	Production Company
**Semirigid Ureteroscope (Fr)**	6/7; 5/9	STORZ
**Ureteral Sheath (Fr)**	10/12; 12/14	ROCAMED
**Reusable Ureteroscope (Fr)**	7	STORZ
**Single-Use Ureteroscope (Fr)**	7.5	PUSEN
**Ureteral Guidewires (cm)**	150	COOK; BOSTON
**Holmium Laser, Fibers (µm)**	272; 365	SPHINX (30W)
**Pulsed Thulium Laser, Fibers (µm)**	272; 365	DORNIER (60W)
**Basket (ZERO TIP) (Fr)**	1.9	BOSTON
**Ureteral Stent (Fr–cm)**	4.8–24; 5–26; 6–26; 7–26	COOK/BOSTON
**Bladder Catheter (Fr)**	18; 20 (Foley)	RUSCH

**Table 2:** Perioperative characteristics of the total cohort.

Fattori	*n* = 113
**Age (years, median, IQR)**	58 (50–66)
**BMI (m^2^/Kg, median, IQR)**	26.12 (23.74–29.39)
**Site** - **Right** - **Left** - **Bilateral**	52 (46%) 58 (51.3%) 3 (2.7%)
**Renal location** - **Not in the kidney superior calix** - **Medium calix** - **Inferior calix** - **Renal pelvis** - **Ureteropelvic junction**	41 (36.28%) 11 (9.73%) 14 (12.39%) 11 (9.73%) 22 (19.47%) 14 (12.39%)
**Ureteral location** - **Not in ureter** - **Upper ureter** - **Medium ureter** - **Lower ureter**	66 (58.41%) 22 (19.47%) 11 (9.73%) 14 (12.39%)
**Maximum diameter (mm, median, IQR)**	12 (9.5–17.5)
**Number of calculi** - **1** - **2** - **>2** - **Staghorn Calculus**	79 (69.91%) 16 (14.16%) 11 (9.73%) 7 (6.19%)
**Hounsfield Unit (median, IQR)**	1350 (950–1640)
**Preoperative hydronephrosis** - **Yes** - **No**	30 (26.5%) 83 (73.5%)
**Preoperative ureteral stenting** - **Yes** - **No**	21 (18.6%) 92 (81.4%)
**Preoperative white cell count (median, IQR)**	8.27 (6.49–10.32)
**Preoperative creatinine (mg/dL, median, IQR)**	1.21 (1.02–2.16)
**Preoperative urine culture**	0 (0%)
**Postoperative white cell count (median, IQR)**	9.41 (8.16–11.36)
**Postperative creatinine** **(mg/dL, median, IQR)**	1.18 (1.08–1.27)
- **Postoperative urine culture negative** - **Positive**	46 (40.7%) 67 (59.3%)
**Type of procedure** - **RIRS** - **ULT** - **PCNL** - **ECIRS**	54 (47.8%) 52 (46.1%) 4 (3.5%) 3 (2.6%)
**Type of ureteroscope** - **Flexible** - **Semi-rigid**	36 (31.8%) 77 (68.2%)
**Type of instrument** - **Disposable** - **Reusable**	31 (27.4%) 82 (72.6%)
**Type of energy** - **Holmium** - **Super Thulium** - **Trilogy**	75 (66.4%) 34 (30.1%) 4 (3.5%)
**Postoperative ureteral stenting**	113 (100%)
**Operatory time (min, median, IQR)**	57 (35–85)

**Table 3:** Univariable and multivariable logistic regression analyses.

Variable	Univariate Analysis					Multivariate Analysis		
		95.0% CI				95.0% CI		
	Odds Ratio	Inferior	Superior	*p*-Value	Odds Ratio	Inferior	Superior	*p*-Value
**Age**	0.99	0.96	1.02	0.59	-	-	-	-
**BMI**	1.01	0.92	1.08	0.9	-	-	-	-
**Side**	0.78	0.39	1.56	0.49	-	-	-	-
**Position (related to kidney)**	1.1	0.9	1.35	0.32	-	-	-	-
**Position (related to ureter)**	0.85	0.59	1.21	0.36	-	-	-	-
**Maximum diameter**	1.15	1.05	1.25	**0.001**	0.98	0.83	1.16	0.88
**Number of stones**	2.24	1.25	4.01	**0.006**	0.9	0.3	2.68	0.85
**Hounsfield Units**	0.99	0.99	1	0.21	-	-	-	-
**Preoperative white cell count**	1.08	0.94	1.23	0.26	-	-	-	-
**Preoperative-creatinine**	1.05	0.94	1.18	0.33	-	-	-	-
**Postoperative white cell count**	1.08	0.95	1.23	0.2	-	-	-	-
**Postoperative s-creatinine**	1.03	0.89	1.19	0.64	-	-	-	-
**Type of instrument (multi-use vs. single-use)**	**1.28**	**0.55**	**0.97**	**0.01**	**1.3**	**0.55**	**0.99**	**0.03**
**Type of energy**	0.89	0.45	1.76	0.75	-	-	-	-
**Operative time**	**1.13**	**1.08**	**1.18**	**<0.001**	**1.14**	**1.08**	**1.2**	**<0.001**

## Data Availability

The data presented in this study are available on request from the corresponding author.
